# Membrane interaction and disulphide-bridge formation in the unconventional secretion of Tau

**DOI:** 10.1042/BSR20210148

**Published:** 2021-08-05

**Authors:** Marianna Hellén, Arnab Bhattacharjee, Riikka-Liisa Uronen, Henri J. Huttunen

**Affiliations:** Neuroscience Center, HiLIFE, University of Helsinki, Helsinki 00014, Finland

**Keywords:** Alzheimers disease, Tauopathy, unconventional secretion

## Abstract

Misfolded, pathological tau protein propagates from cell to cell causing neuronal degeneration in Alzheimer’s disease and other tauopathies. The molecular mechanisms of this process have remained elusive. Unconventional secretion of tau takes place via several different routes, including direct penetration through the plasma membrane. Here, we show that tau secretion requires membrane interaction via disulphide bridge formation. Mutating residues that reduce tau interaction with membranes or formation of disulphide bridges decrease both tau secretion from cells, and penetration through artificial lipid membranes. Our results demonstrate that tau is indeed able to penetrate protein-free membranes in a process independent of active cellular processes and that both membrane interaction and disulphide bridge formation are needed for this process. QUARK-based *de novo* modelling of the second and third microtubule-binding repeat domains (MTBDs), in which the two cysteine residues of 4R isoforms of tau are located, supports the concept that this region of tau could form transient amphipathic helices for membrane interaction.

## Introduction

The intracellular accumulation of misfolded, hyperphosphorylated tau in soluble oligomers, insoluble paired helical filaments and neurofibrillary tangles (NFTs), is a neuropathological hallmark of tauopathies, a group of neurodegenerative diseases, including Alzheimer’s disease [1]. Under normal conditions tau, mainly expressed in neurons, binds to microtubules and promotes their assembly. In pathological conditions tau detaches from microtubules, becomes hyperphosphorylated and misfolded, finally forming pathological tau aggregates in the cytosol [2]. Tau pathology can spread in brain between neuroanatomically connected regions by cell-to-cell transmission where pathological tau species are secreted by affected neurons and internalised by adjacent cells, where they can template misfolding of healthy tau [2,3]. The molecular pathways used by pathological tau to spread between cells may present a new druggable target to slow down progression of tau pathology and are therefore of extraordinary interest but remain incompletely understood.

Tau is a cytosolic natively unfolded protein that does not have a stable secondary structure in solution, but has been suggested to undergo context-dependent folding and adopt transient secondary structures upon binding to either microtubules, F-actin or lipid membranes [4–8]. Tau, or shorter fragments including the microtubule-binding repeat domains (MTBDs), can be secreted from cells via multiple unconventional routes, both vesicular and non-vesicular [9]. We and others have shown that one of tau’s secretion routes takes place directly through the plasma membrane [10,11]. Unconventional protein secretion (UPS) is a group of cellular routes that guide proteins to the extracellular space bypassing the traditional ER-Golgi pathway. UPS is divided into four types, type 1 being direct translocation through the PM [12]. The best characterised protein using this pathway is Fibroblast Growth Factor 2 (FGF2) [13]. Interestingly, the direct translocation of tau across the plasma membrane appears to share some mechanistic features with the unconventional secretion mechanism of FGF2. We and others have shown that secretion of both proteins requires the presence of phosphatidyl inositol (4,5) bisphosphate (PIP_2_) and heparan sulphate proteoglycans on the PM [10,11]. While no homology exists between these proteins, the intriguing similarities in their mode of secretion encouraged us study this subject further. Unlike tau, however, FGF2 has a characteristic tertiary structure [14,15], and apparently some of the FGF2 surface-located features regulate the recruitment of FGF2 to the inner leaflet of plasma membrane to initiate the secretion process. Muller et al. explored the role of two unique surface-exposed cysteine residues (Cys^77^ and Cys^95^) in FGF2 (absent from the other FGF family proteins) in the process of unconventional secretion of FGF2 [16]. Mutation of these Cys residues to Ala impairs secretion of FGF2 whereas introduction of these Cys residues in FGF4 results in unconventional secretion of FGF4 (an otherwise absent characteristic from FGF4). While tau appears to share some similarities with unconventional secretion of FGF2, such as the interaction with specific inner leaflet lipids and requirement of outer leaflet HSPGs for secretion, the mechanistic details of the secretion process remain unknown. The MTBDs of tau have been proposed to bind to membranes via formation of amphipathic helices [4] and specific valine residues located in this region, in close vicinity of the two Cys residues present in the 4R isoforms of tau, were reported to play an important role in tau’s lipid interactions [5]. Here, we combined *in silico* modelling and cell biological assays to test the hypothesis that the two cysteine residues present in the MTBD of tau or the membrane binding-associated valines play a similar role in the secretion process as they do in unconventional secretion of FGF2.

## Materials and methods

### Plasmid constructs

The cDNA for wildtype tau (human isoform 0N4R) was purchased from Thermo Scientific, and cDNAs containing mutations V287E, K311A, V318E (tau-MBD) and C291A and C322A (tau-C291/322A) were synthesised by GenScript. The original phGluc plasmids were donated by Prof. Stephen Michnick (University of Montreal). The hGluc fragments were cloned to the C-terminus of tau, tau-MBD and tau-C291/322A in a pcDNA3.1/zeo backbone. All plasmids were sequenced to confirm their identity.

### N2A cell culture and transfection

Mouse Neuro-2a (N2A) neuroblastoma cells (ATCC) were cultured in DMEM (Corning, Lonza) supplemented with 10% (v/v) of foetal bovine serum (Invitrogen), 1% penicillin/streptomycin (Lonza) and 1% l-glutamine (Lonza) at 37°C with 5% CO_2_ and water-saturated air. Transfection of N2A cells with plasmid DNA was performed 24 h after plating using JetPei reagent (Polyplus) according to manufacturer’s instructions.

### Dot blot and Western blot assays

For dot blot and Western blot assays, N2A cells plated on 10-cm plates were transfected with either phGluc2-Tau, phGluc2-Tau-MBD or phGluc2-Tau-C291/322A plasmids. Cells and culture media were collected after 24 h. Dot blot assays were performed as described in [11]. Briefly, 100 µl of conditioned media and 50 µl of cell lysate were applied to a dot blot apparatus (96-well Manifold Spot-Blot unit, Whatman/Sleicher and Schuell) and proteins were trapped by filtration in a methanol-activated PVDF membrane (GE Healthcare). Membranes were probed with tau-5 monoclonal antibody (Invitrogen) and horseradish peroxidase (HRP)-conjugated mouse secondary antibody, the chemiluminescence signal was detected by using ECL Plus Western blotting detection reagent (Thermo Scientific) and Syngene imaging system. Membranes were quantitatively analysed by determining the ratio between extracellular and intracellular proteins with ImageJ software.

For Western blot of cell lysates, equal amounts of total protein (90 µg) per lane were resolved in a 4–20% gradient precast polyacrylamide gels (Mini-Protean TGX, Bio-Rad) under non-reducing conditions. Separated proteins were transferred to a PVDF membranes using Trans-Blot Turbo Midi PVDF Transfer Packs and Trans-Blot Turbo Transfer System (both from Bio-Rad). The membranes were blocked with 5% (w/v) milk in TBST and probed with the tau-5 and β-actin (Sigma) primary antibodies. After incubation of the membranes with HRP-conjugated mouse secondary antibody, the chemiluminescence signal was detected by using ECL Plus Western blotting detection reagent (Thermo Scientific) and Syngene imaging system.

### Immunofluorescence microscopy

N2A cells were grown on glass coverslips coated with Poly-l-lysine (Sigma), transfected either with phGluc1-Tau, phGluc1-TauMBD or phGluc1-Tau-C291/322A plasmids per coverslip and fixed for 20 min with 4% PFA in PBS. After washing with PBS, the cells were incubated for 1 h in blocking buffer (5% goat serum, 1% BSA, 0.1% gelatin, 0.1% Triton X-100, 0.05% Tween-20 in PBS). The cells were incubated overnight at +4°C with tau-5 primary antibody diluted 1:500 in 1% BSA and 0.1% gelatin. After washing with PBS, the cells were incubated for 45 min at room temperature with Alexa Fluor 488-conjugated goat anti-mouse secondary antibody diluted 1:1000 into PBS. The cells were washed with PBS and incubated with Hoechst 33342 (Invitrogen) nuclear stain diluted 1:10000 in PBS for 10 min at room temperature. Finally, after washing with PBS and milli-Q water the coverslips were mounted on microscope slides with Prolong Gold antifade reagent (Invitrogen). Images were taken with a Zeiss LSM710 confocal microscope.

### Preparation of giant unilamellar vesicles

Giant unilamellar vesicles (GUVs) with a plasma membrane inner leaflet-like lipid composition consisting of 30 mol% phosphatidylcholine (PC) (15 mol% DOPC + 15 mol% DPPC), 25 mol% cholesterol (Chol), 10 mol% sphingomyelin (SM), 25 mol% phosphoethanolamine (PE), 10 mol% phosphatidylserine (PS) and 1 mol% rhod-PE (Avanti Polar Lipids) were prepared using the electroformation technique (Vesicle Prep Pro, Nanion Technologies GmbH) according to manufacturer’s instructions. Sixty-seven micrograms of lipids dissolved in chloroform was spread evenly on the conductive side of an indium tin oxide (ITO) slide and air-dried few minutes in the fume hood and the dried lipid film was hydrated by 300 mM sucrose solution. Electroformation was carried out using the following electroswelling parameters: rise time to 1.5 V for 5 min, followed by 50 min of constant amplitude of 1.5 V and fall time 25 min, at a constant frequency of 10 Hz at 45°C. The prepared GUVs in sucrose solution were collected from the ITO slide and stored at +4°C.

### Imaging and quantification of membrane translocation of tau peptides using GUVs

For monitoring tau translocation into the lumen of GUVs fluorescein (FITC)-tagged (N-terminus) wildtype tau peptide (residues 275–325 according to the human tau isoform 2N4R numbering), tau-MBD peptide (residues 275–325 with V287E, K311A and V318E mutations) and tau-C291/322A peptide were used. Peptides were manufactured by Apeptide Shanghai and their identity confirmed by mass spectrometry. For monitoring the formation of membrane pores, a small fluorescent tracer (Alexa647) was used. A total of 48–58 μl of GUVs, 20 µM tau peptides and 2 µM Alexa647 were incubated in rotating mixer at room temperature for 24 h with PBS (total volume of mixture 100 μl). Images were recorded at RT with a Zeiss LSM710 confocal microscope and analysed with ImageJ software by using the Radial profile plugin. Fluorescence of FITC-tau and Alexa 647 were measured as a function of distance from the GUV-membrane indicated by Rhodamine-PE. FITC-tau and Alexa647 values were normalised to average value outside GUVs.

For DTT experiments, wildtype tau peptide was preincubated for 1 h at RT in rotating mixer with DTT. After preincubation, GUVs (38–48 μl) and Alexa647 were added to the preincubation mixture. The final incubation mixture (total volume 100 µl), including 20 µM tau peptide, 100 mM DTT and 2 µM Alexa647 was incubated for 24 h at RT in a rotating mixer before imaging as described above.

### Statistical analyses

Differences between multiple means were tested by Welch’s ANOVA followed by Dunnett’s post-test. In the dot blot experiment, ‘*n*’ represents the number of independent replications. In the GUV experiment, ‘*n*’ represents individual GUVs in a given peptide condition. All values were expressed as mean ± SEM. All *P*-values below 0.05 were considered significant. *P<0.05, **P<0.01, ****P<0.0001.

## Results

### *Ab initio* modelling of tau MTBDs suggest folding into α-helical structures with amphipathic nature

**Figure 1:**
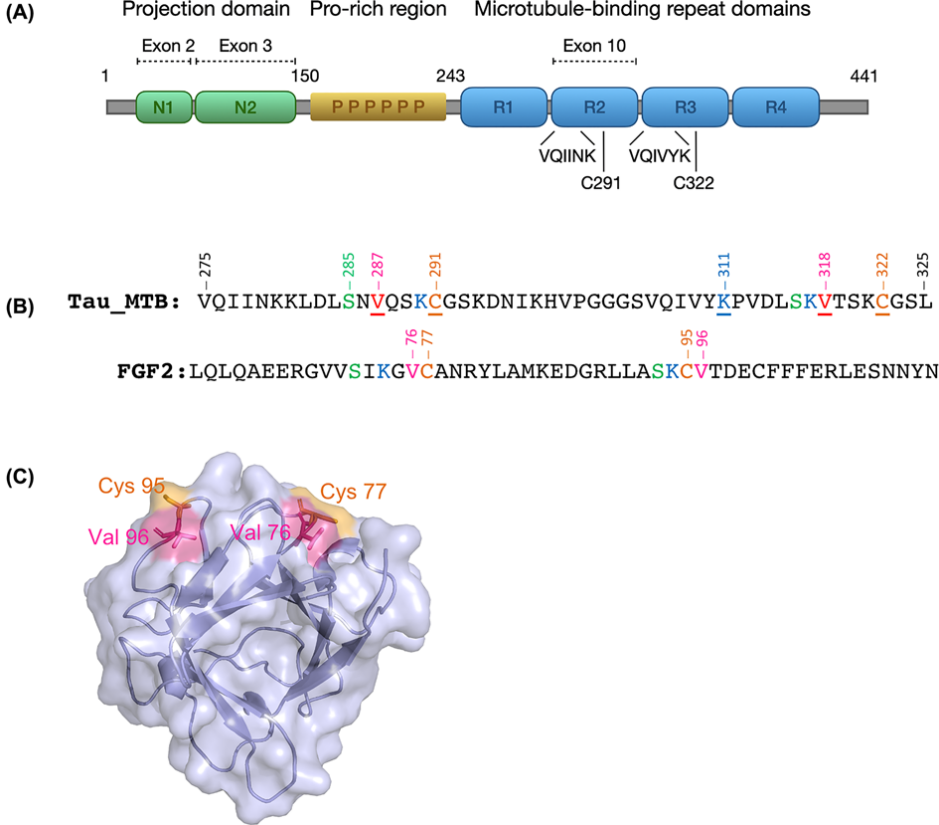
Location of cysteines in the tau MTBDs 2 and 3 and FGF2 (**A**) The domain structure of the longest (2N4R) isoform of human tau protein. Location of the projection domain, proline-rich domain, MTBDs, the two hexapeptides and the two cysteines are shown. (**B**) Sequences of tau MTB Zone and FGF2. The cysteine containing repeats are highlighted in yellow. Residues in the vicinity of the Cys are coloured using the same colour scheme (K-Blue; V-Pink; S-Green; C-Orange; S-Green). The residues mutated in the present study are underlined. (**C**) A surface representation of FGF2 X-ray structure (PDB: 1BFF) showed in a translucent mode with the cartoon ray traces. Cys^77^ and Cys^95^ are shown in orange and the adjacent valines in pink.

The 4R isoforms of tau contain two Cys residues, Cys^291^ and Cys^322^, located in the MTBDs two and three, respectively (Figure 1A). Disulphide-bridged tau dimers have been found in extracellular space and it was suggested that intermolecular disulphide bridging provides structural stability for tau dimers that could favour propagation of tau pathology [17]. As shown in Figure 1B, the regions surrounding the Cys residues in FGF2 and tau are composed of similar amino acids, but there is no obvious shared sequence motif present. Interestingly, the two Val residues (Val^287^ and Val^318^) located close to the Cys^291^ and Cys^322^ residues were found to be critical for the ability of tau to bind to phospholipids [5], indicating that the MTBD regions near the Cys residues are involved in tau’s lipid/membrane interactions.

**Figure 2:**
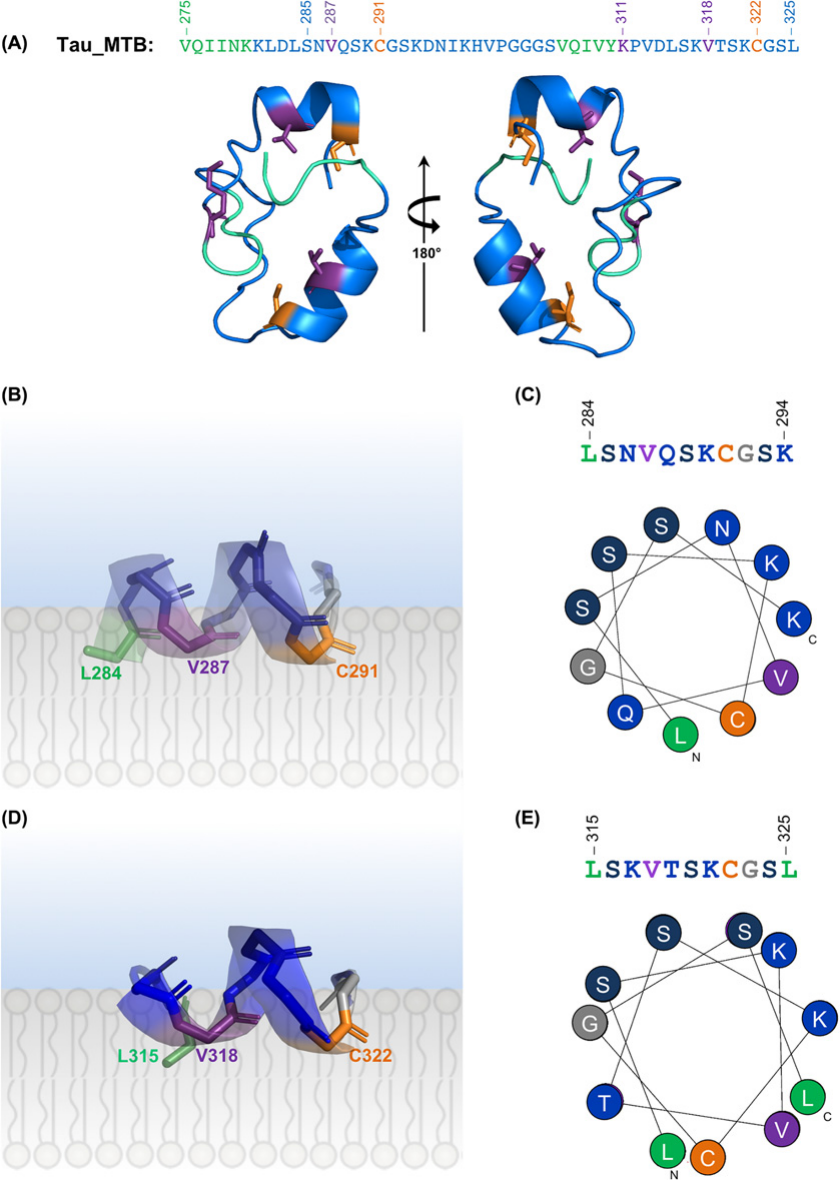
A *de novo* model of tau cysteine-containing microtubule-binding area (275–325) (**A**) A cartoon representation of the tau *de novo* model (shown with 180° rotation) obtained using QUARK. The tau sequence, corresponding to the second and third MTBDs, is shown using the same colour scheme. (**B,C**) A 3D cartoon representation (B) and helical wheel diagram (C) of the Cys^291^ containing α-helix with a theoretical visualisation at the membrane interface. Leu^284^, Val^287^ and Cys^291^ are localised on the same side of the helix. (**D,E**) A 3D cartoon representation (D) and helical wheel diagram (E) of the Cys^322^ containing α-helix with a theoretical visualisation at the membrane interface. Leu^315^, Val^318^ and Cys^322^ are localised on the same side of the helix.

As shown in Figure 1C, the Cys^77^ and Cys^95^ of FGF2 are located on the surface of the protein with a distance that would not allow intramolecular disulphide bridging. There is a conserved Val residue located next to both Cys residues in a protruding loop that could be embedded in the membrane upon recruitment to the inner leaflet of the plasma membrane. We used *ab initio* modelling [18] to explore if the regions near the Cys residues in tau (focusing on residues 275–325) could be prone to transient adoption of secondary structure as a previous study suggested that the tau MTBDs may adopt amphipathic helices upon membrane interaction [4]. As shown in Figure 2A, theoretical computational modelling suggests that the regions surrounding the Cys residues of tau could fold into α-helical structures as suggested previously [5]. Interestingly, in both cases the Cys residue is located at the end of a putative α-helix. Importantly, the Cys^291^ and Val^287^ in helix 1 (Figure 2B,C) and Cys^322^ and Val^318^ in helix 2 (Figure 2D,E) are located on the same side of a helix which could favour simultaneous membrane embedding, similar to FGF2. Moreover, the *ab initio* models support the previous studies that suggested the presence of amphipathic helices in tau that are capable of membrane interaction [4,5].

In order to test the role of the Cys and Val residues in the membrane interaction and secretion of tau, we generated tau (0N4R) expression plasmids and 51-aa synthetic peptides containing the hexapeptide repeats (VQIINK and VQIVYK) and the regions surrounding the Cys residues, as well full-length tau (4R) with either C291A/C322A mutations (tau-C291/322A), or V287E/K311A/V318E mutations (tau-MBD) (Figure 1B). These mutants were compared with wildtype tau in cell-free GUV membrane interaction experiments (peptides) and a cell-based tau secretion assay (expression plasmids).

### Tau secretion from cells requires membrane interaction and disulphide bridges

**Figure 3:**
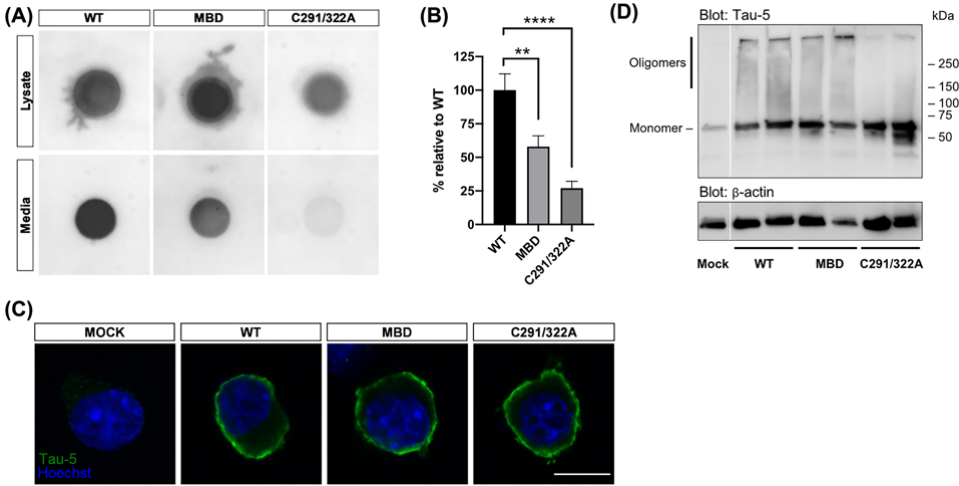
Tau secretion is reduced by tau-MBD and tau-C291/322A mutations in N2A cells (**A**) A representative dot blot image of cell lysate and conditioned media from N2A cells transfected with wildtype tau, tau-MBD mutant or tau-C291/322A mutant, stained with tau-5 antibody. (**B**) Quantification of the densitometric dot blot data (*n*=6). Conditioned media dots were normalised by the corresponding cell lysate dots. Data are represented as mean ± SEM. **P<0.01, ****P<0.0001 (Welch’s ANOVA). (**C**) Confocal micrographs of N2A cells transfected with wildtype or mutant tau (tau-MBD or tau-C291/322A). Representative images of tau-5 immunostaining with Hoechst 33342 nuclear counterstain are shown. Scale bar, 10 µm. (**D**) Western blot analysis of N2A cells transfected with wildtype or mutant tau (tau-MBD or tau-C291/322A), stained with tau-5 antibody. β-actin was used as a loading control.

To test if tau-C291/322A and tau-MBD mutations affect their secretion from cells, we overexpressed human tau (isoform 0N4R) with and without these mutations in N2A cells and measured tau in cells and media using dot blot. The secretion of both tau mutants from N2A cells were decreased compared with wildtype tau (Figure 3A,B). The reduction in tau-MBD secretion was 42 ± 8% compared with wildtype tau, indicating that the three residues previously indicated in lipid-binding [5] are indeed necessary for tau secretion, and further suggesting that interaction of tau with membranes is required for its secretion. The tau-C291/322A mutant was secreted 73 ± 5% less effectively compared with wildtype tau (Figure 3B), showing that the two Cys residues play an important role in tau secretion, similar to FGF2. These results suggest that both plasma membrane interaction of tau by these MTBD-located residues and the capability of tau to form intra- and/or intermolecular disulphide bridges are key determinants of tau secretion from cells. To test if these mutants are unable to reach the plasma membrane (due to e.g. defective transport to plasma membrane), we stained tau overexpressing N2A cells with tau-5 antibody. There was no difference in tau staining pattern in cells expressing the mutant tau constructs, indicating that membrane interaction, rather than transport to the plasma membrane causes the defect in secretion of these mutants forms of tau (Figure 3C). To test if tau-C291/322A and tau-MBD mutations decrease the oligomerisation properties of tau, cell lysates were resolved in non-reducing conditions and analysed by Western blot. All tau constructs showed a strong band corresponding to monomeric tau while in cells expressing wildtype tau and tau-MBD there were clearly more insoluble tau trapped in the stacking gel as well as a high molecular weight smear of soluble tau oligomers than in cells expressing the tau-C291/322A mutant (Figure 3D). These results suggest that tau-C291/322A mutant has decreased propensity to form high molecular weight oligomers compared with wildtype tau and the tau-MBD mutant.

### Tau penetration of artificial membranes requires membrane interaction and disulphide bridging

**Figure 4:**
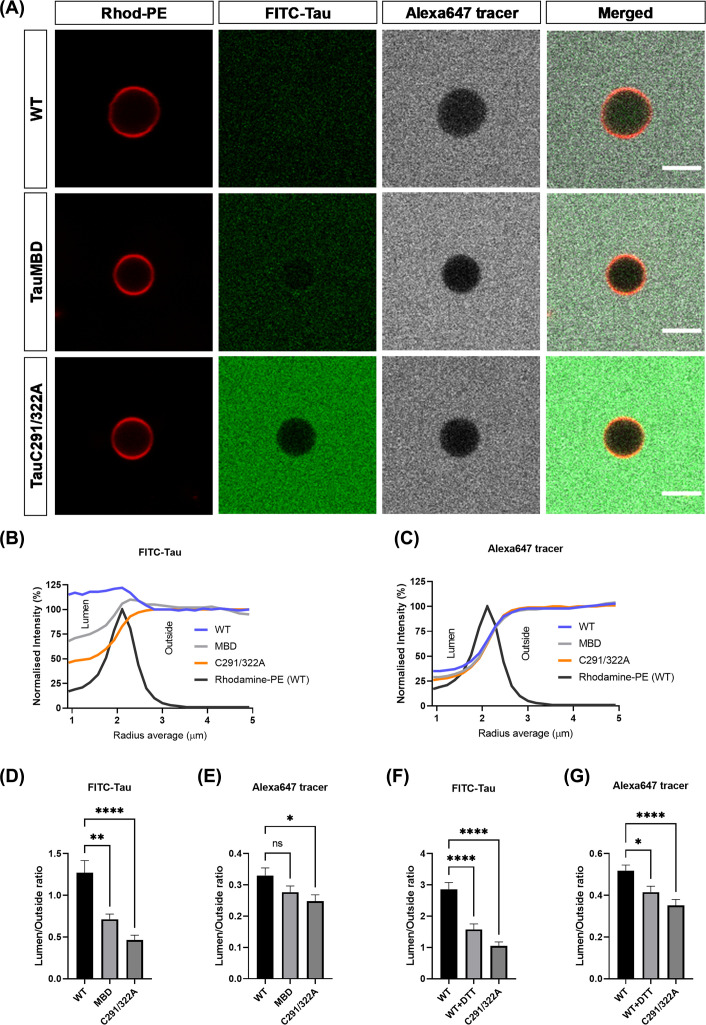
Interaction of wildtype and mutant tau peptides with GUVs (**A**) Confocal micrographs of GUVs incubated 24 h with FITC-tagged wildtype or mutant (tau-MBD or tau-C291/322A) tau peptides (20 µM) and Alexa647 (2 μM). Rhodamine-PE was incorporated into GUV preparation as a membrane marker. Scale bar 5 µM. Average profile plots for FITC-tau (**B**) and Alexa647 (**C**) for all tau peptides in addition to average Rhodamine-PE values for wild type peptide to indicate the position of the GUV membrane. (**D,E**) Quantitative analysis of membrane translocation (D) and pore formation (E) of wildtype and mutant tau (tau-MBD or tau-C291/322A) peptides. Data were derived from the same experimental GUVs as in panels (B,C). Data are presented as mean ± SEM. *P<0.05, **P<0.01, ****P<0.0001 (Welch’s ANOVA). For each tau peptide 95–99 individual GUVs were analysed. (**F,G**) Membrane translocation (F) and pore formation (G) of wildtype tau, DTT-treated wildtype tau and tau-C291/322A peptides diluted in PBS and preincubated 1 h with or without DTT before addition of GUVs and Alexa647 and final 24-h incubation. Data are presented as mean ± SEM. *P<0.05, ****P<0.0001 (Welch’s ANOVA). For each tau peptide condition 103–112 individual GUVs were analysed.

To study tau interaction with lipid membranes in more detail in a simpler cell-free system where fine control of membrane components is possible, we prepared GUVs with an inner plasma membrane leaflet-mimicking lipid composition. We incubated small (51 aa) FITC-tagged tau peptides with GUVs for 24 h, together with free Alexa 647 dye to visualise: (1) tau penetration into GUVs and (2) Alexa 647 penetration into GUVs as a proxy for tau-generated pores in the membrane, as similar experiments have revealed details of secretion of FGF2 [19]. GUVs incubated with wt tau peptide showed tau penetration into the lumen of GUVs (Figure 4A,B), indicating that indeed, Tau is able to penetrate lipid membranes in the absence of active cellular processes. While some Alexa 647 was found inside GUVs, it did not extensively penetrate the GUV membrane, and was mostly found outside GUVs, indicating that while tau membrane penetration shares some characteristics with FGF2, it is not completely similar (Figure 4A,C).

We next mutated key residues in tau peptides (Figure 1B) to test if membrane interaction and cysteines are required for tau membrane penetration. Compared with GUVs incubated with wt tau, GUVs incubated with tau-MBD showed decreased penetration into the lumen of GUVs (Figure 4A,B), confirming that the residues previously implicated in phospholipid binding of tau are indeed required for membrane interaction and penetration.

As shown in Figure 4A,B, also tau-C291/322A showed decreased penetration into the lumen of GUVs compared with wildtype tau, and as shown in Figure 4B, this decrease in luminal penetration was more prominent for tau-C291/322A than for tau-MBD when compared with wildtype tau. These results suggest that the presence of cysteines in the membrane interacting part of tau facilitate the membrane penetration process, possibly through generation of disulphide cross-linked dimers.

The average lumen/outside fluorescence ratios for each peptide are shown in Figure 4D and those results verify our notion that the luminal penetration of both tau mutants (MBD and C291/322A) is significantly decreased compared with wildtype tau and that this decrease is more prominent for double cysteine-mutant tau. In case of Alexa 647 (Figure 4E), a similar statistical analysis showed significant difference only between wildtype and double cysteine-mutated tau suggesting that during the luminal penetration process of wildtype tau the GUV membrane becomes more permeable for Alexa 647.

Finally, we tested the role disulphide bridge formation in the tau membrane penetration process by preincubating the FITC-tagged wildtype tau with a high concentration of DTT before addition of GUVs. The final concentration of DTT after addition of GUVs was 100 mM which ensured the highly reducing environment for the whole duration of the assay (∼24 h). The preincubation of wildtype tau with DTT decreased the luminal penetration of tau into the GUVs significantly as shown in Figure 4F. Notably, in the presence of DTT also tau-induced membrane permeability to Alexa 647 was decreased (Figure 4G).

## Discussion

We [11] and others [10] have previously shown that tau can be secreted from cells via an unconventional secretion route directly through the membrane. Katsinelos et al. [10] suggested that recruitment of tau to the membrane occurs via tau binding to PIP_2_, and we showed that changing membrane properties in cells to more fluid (by decreasing Chol or sphingomyelin), tau secretion is inhibited. However, the means of tau membrane binding and penetration remained elusive. Here, we studied this question further. We were intrigued by Ait-Bouziad et al. [5] who showed that tau can indeed interact with lipid membranes via specific residues in the MTBDs and that tau’s structure changes upon binding to membranes. The tau protein in complex with phospholipids was noticed to have structural similarity with tau found in the development of AD pathology. The role of the R2 domain of tau in formation of tau-phospholipid complexes was also reported by the same group [5].

For type I unconventional secretion, tau would need to bind to and eventually penetrate into the membrane. Interestingly, several studies have suggested that tau oligomers can form membrane pores or ion-permeable channels in membranes [20,21]. We showed that the residues reported to be important for membrane binding (V287E, K311A, V318E) [5] are also important for tau secretion. We also demonstrated that these residues are important for membrane binding and penetration. Moreover, we showed that the two Cys residues located near the hexapeptides (VQIINK in R2 and VQIVYK in R3; Figure 1A) in the MTBDs, that are centrally involved in tau aggregation, play a key role in membrane interaction and secretion tau. This is in-line with a previous report suggesting that covalent disulphide-mediated dimerisation plays an important role in the tau secretion process [17]. Interestingly, while this paper was in review, a report was published showing that the two cysteine residues are a critical *cis*‐element in unconventional secretion of tau [22].

FGF2, a secreted growth factor, utilises direct PM translocation to exit cells, forms disulphide bridges with its surface exposed cysteines upon binding to membranes and depends on them for secretion. Mechanistically, there are several similarities between FGF2 and tau secretion: PIP_2_-mediated membrane recruitment, facilitation by disulphide bridging and external leaflet HSPGs (reviewed by [9]). While FGF2 has an established globular tertiary structure, tau is considered to be a natively unfolded protein. However, a number of reports have suggested that tau may undergo context-dependent folding [4,8]. Importantly, the MTB region containing the residues shown in this study, and previously by Ait-Bouziad et al. [5], were suggested to adopt amphipathic helices upon membrane binding [4]. Our *ab initio* modelling experiments suggested that the tau sequence containing the R2 and R3 domains indeed can form helical secondary structure with the key residues Val^287^, Cys^291^, Val^318^ and Cys^322^ positioned in a putative membrane interface (Figure 2). Here, *ab initio* modelling of the MTBDs was used for two reasons: there were no fitting template to be used for homology modelling, and to keep the bias of the structure of the globular proteins out of the way by using a homology modelling approach. We wanted to understand all of the possible structural conformations which tau MTB region could adopt in the presence of the membrane, which was not used for solving the NMR structure (PDB: 2mz7) [8] and we felt that an *ab initio* model (though done in absence of a membrane) might shed new light on this question. The *ab initio* model is a theoretical tool and cannot fully elucidate the tau conformation in presence of a membrane, but it can take us one step closer to understanding transient context-dependent folding of tau upon membrane interaction and its role in the unconventional secretion of tau.

Here we showed that tau secretion and its interaction with membranes with inner leaflet properties is dependent on residues involved in disulphide bridge formation and lipid binding. Our *ab initio* modelling, cell-free membrane interaction and cell-based tau secretion methods have some limitations and further studies are needed to investigate tau–membrane interaction in other experimental systems including complex biological membranes. Moreover, molecular dynamics simulation could shed more light on dynamic aspects of tau folding upon membrane interaction and its penetration to the membrane. As tau secretion is a critical part of the pathological propagation and disease progression processes, better understanding of the molecular events underlying this phenomenon has important ramifications for understanding pathobiology of tauopathies and development of novel therapeutic approaches.

## Data Availability

The data that support the findings of the present study are available from the corresponding author upon reasonable request.

## Abbreviations

AD: Alzheimer's disease
Chol: cholesterol
ER: endoplasmic reticulum
FGF2: fibroblast growth factor 2
GUV: giant unilamellar vesicle
HRP: horseradish peroxidase
HSPG: heparan sulphate proteoglycan
ITO: indium tin oxide
MTBD: microtubule-binding repeat domain
N2A: Neuro-2a
PFA: paraformaldehyde
PIP_2_: phosphatidyl inositol (4,5) bisphosphate
PM: plasma membrane
UPS: unconventional protein secretion
